# Integrated miRNA-mRNA analysis reveals regulatory pathways underlying the curly fleece trait in Chinese tan sheep

**DOI:** 10.1186/s12864-018-4736-4

**Published:** 2018-05-11

**Authors:** Yufang Liu, Jibin Zhang, Qiao Xu, Xiaolong Kang, Kejun Wang, Keliang Wu, Meiying Fang

**Affiliations:** 1 Department of Animal Genetics and Breeding, National Engineering Laboratory for Animal Breeding, MOA Laboratory of Animal Genetics and Breeding, College of Animal Science and Technology, China Agricultural University, No. 2 Yuanmingyuan West Rd, 100194 Beijing, People’s Republic of China; 2 College of Life Sciences and Food Engineering, Hebei University of Engineering, Handan, 056021 People’s Republic of China; 3 College of Agriculture, Ningxia University, Yinchuan, 750021 People’s Republic of China; 4 Department of Cell and Molecular Biology, Beckman Research Institute of the City of Hope, Duarte, CA 91010 USA; 5 Beijing Key Laboratory for Animal Genetic Improvement, Beijing, 100193 People’s Republic of China

**Keywords:** Tan sheep, miRNA-mRNA analysis, Curly fleece, Regulatory network, *KRT83*, *miR-432*

## Abstract

**Background:**

Tan sheep is an indigenous Chinese breed well known for its beautiful curly fleece. One prominent breed characteristic of this sheep breed is that the degree of curliness differs markedly between lambs and adults, but the molecular mechanisms regulating the shift are still not well understood. In this study, we identified 49 differentially expressed (DE) microRNAs (miRNAs) between Tan sheep at the two stages through miRNA-seq, and combined the data with that in our earlier Suppression Subtractive Hybridization cDNA (SSH) library study to elucidate the mechanisms underlying curly fleece formation.

**Results:**

Thirty-six potential miRNA-mRNA target pairs were identified using computational methods, including 25 DE miRNAs and 10 DE genes involved in the MAPK signaling pathway, steroid biosynthesis and metabolic pathways. With the differential expressions between lambs and adults confirmed by qRT-PCR, some miRNAs were already annotated in the genome, but some were novel miRNAs. Inhibition of *KRT83* expression by *miR-432* was confirmed by both gene knockdown with siRNA and overexpression, which was consistent with the miRNAs and targets prediction results.

**Conclusion:**

Our study represents the comprehensive analysis of mRNA and miRNA in Tan sheep and offers detailed insight into the development of curly fleece as well as the potential mechanisms controlling curly hair formation in humans.

**Electronic supplementary material:**

The online version of this article (10.1186/s12864-018-4736-4) contains supplementary material, which is available to authorized users.

## Background

Chinese Tan sheep (*Ovis aries*) are indigenous to Mongolian plateau and are usually found in the Ningxia Hui Autonomous Region and Gansu province in China. The economic importance of the Tan breed is mainly attributed to the high-quality pelts from one-month-old lambs. These pelts, characterized by lustrous curly fleece of a natural white color after processing, are thin, light, and widely used for the production of luxury apparels. However, the curly fleece always disappears gradually as the lambs mature into adulthood.

The developmental regulation of curly fleece or hair in mammals is highly complicated. Curliness is programmed within the hair follicle and determined by the distributions of different types of hair keratins as well as different cell types within the hair [1–3]. In humans, hair fibers vary considerably from very straight hair to tightly sprung coil. Strong association has been revealed between genetic polymorphisms in candidate genes and the curly hair traits by genome-wide association study, but the complete regulatory mechanism underlying varied hair curl is still unclear [4]. In mammals, specific miRNAs are important regulators, implicated in the maintenance of the pluripotent cell state during early embryogenesis, and are thought to play important roles in tissue-specific or organ-specific development [5, 6]. MiRNAs are critical post-transcriptional regulators of hair follicle growth [7–9] that regulate gene expression through the RNA interference pathway and are involved in skin development and cell differentiation. By modulating miRNA abundance, it is possible to fine-tune the expression of proteins within the cells in a very precise manner [10]. Experiments in humans and mice have examined specific miRNAs and their roles in the regulation of the hair follicle cycle [11, 12]. Curly fleece formation at multiple levels, such as post-transcription and translation [13, 14]. Studies related to Chinese Tan sheep initially focused on genetic evaluation and breed development [15]. Researchers subsequently studied phenotypic variations related to wool color, length, density and shape between Chinese Tan sheep and other breeds [16], and concluded that many candidate genes could be used in molecular marker-assisted selection to improve fleece curvature in Tan lambs [17, 18]. However, the regulatory mechanisms associated with these genes remained unclear.

In this study, our aim is to elucidate the mechanisms of curly fleece formation. We presented an integrative analysis combining the SSH cDNA library data generated in our previous study [19] and a new global survey of miRNAs extracted from shoulder skin samples of one-month-old Tan lambs exhibiting curly fleeceand 48-month-old adults exhibiting non-curling fleece. By comparing the observed expression patterns between the two groups, we identified DE miRNAs. An analysis of potential miRNA/mRNA interactions revealed key miRNA/mRNA interacting pairs that correlate with curly hair development. Our findings suggest that changing levels of skin-specific miRNAs, and miRNA interactions with specific gene targets, are involved in regulation of curly fleece development. This report offers deeper insight into the molecular mechanisms underlying the curly hair/fleece trait in Chinese Tan sheep, and demonstrates the power of an integrated analysis that combines global mRNA and miRNA sequence analyses. The results will also aid in the understanding of the curly hair development in humans.

## Results

### Sequencing and analysis of miRNAs obtained from tan sheep

**Fig. 1:**
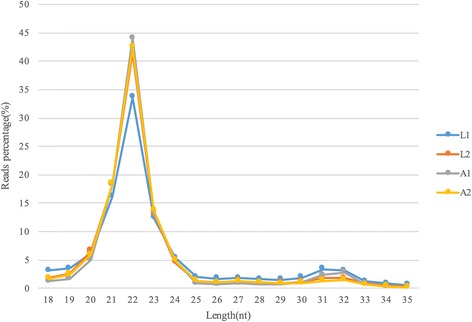
Length distribution of miRNA reads. RNAs were obtained from shoulder skin tissues of 1-month-old lambs (L) and 48-month-old adults (A)

In order to study changes in global miRNA expression related to the distinct wool phenotypes exhibited by Chinese Tan lambs (curly fleece) and adults (non-curling fleece), we obtained shoulder skin tissue samples from two animals in each age group. Total RNA was extracted with TRIzol and an animal total RNA extraction kit (Tiangen, Beijing) as per the manufacturers’ directions and two datasets were generated from the lamb (L1, L2) and adult (A1, A2) samples using Small RNA Sample Pre Kit (Novogene, Beijing, China). Reads of 140~ 160 bp obtained from illumina HiSeq™2500/MiSeq. The reads containing poly-N, 5′ adapter contaminants, and poly- A or T or G or C were filtered out with NGQC(Novogene), together with those without a 3′ adapter or insert tag, and reads with low quality scores. Clean reads after filtering accounted for about 97% of the total raw reads (Table 1). We selected clean reads with length 18–35 nt for subsequent analysis. The length distribution of this subset peaked at 22 nt (Fig. 1). The Pearson correlation between the two individuals in each group was up to 0.96 as calculated from miRNA-seq data, indicating a high concordance rate. The small RNA tags were mapped to the sheep reference genome (ftp://ftp.ensembl.org/pub/release-76/fasta/ovis_aries/dna) using Bowtie [20] with no mismatches permitted, and the locations were used to identify known miRNAs. Novel miRNAs were predicted using the applications miREvo [21] and mirdeep2 [22]. Two hundred and thirty two unique miRNAs including 141 conserved miRNAs and 91 novel miRNAs were identified and assigned to genomic coordinates (Additional file 1: Table S1).

**Table 1:** miRNA-seq quality control statistics

Sample	Read category	Number	Percentage
L1	total reads	12,341,536	100.00%
	N% > 10%	0	0.00
	low quality	7942	0.06
	5′ adapter contamination	1153	0.01%
	3′ adapter null or insert null	280,760	2.27%
	containing polyA/T/G/C	10,394	0.08%
	clean reads	12,041,287	97.57%
L2	total_reads	11,037,839	100.00%
	N% > 10%	0	0.00
	low quality	8657	0.08
	5_adapter_contamine	559	0.01%
	3_adapter_null or insert_null	353,963	3.21%
	with polyA/T/G/C	6319	0.06%
	clean reads	10,668,341	96.65%
A1	total_reads	12,691,784	100.00%
	N% > 10%	0	0.00
	low quality	8732	0.07
	5_adapter_contamine	452	0.00%
	3_adapter_null or insert_null	266,774	2.10%
	with polyA/T/G/C	5719	0.05%
	clean reads	12,410,107	97.78%
A2	total_reads	12,926,796	100.00%
	N% > 10%	0	0.00
	low quality	9846	0.08
	5_adapter_contamine	691	0.01%
	3_adapter_null or insert_null	290,474	2.25%
	with polyA/T/G/C	9115	0.07%
	clean reads	12,616,670	97.60%

### Analysis of conserved and novel miRNAs

**Fig. 2:**
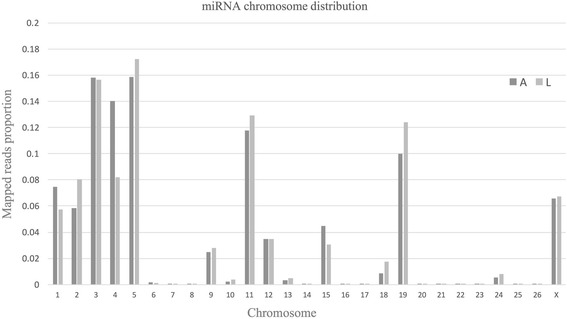
Proportions of mapped miRNAs in each chromosome. miRNA reads were mapped to the sheep reference genome (oar_v3.1). For each chromosome, the average fraction of reads corresponding to lamb (L) and adult samples (A) is shown separately

Hairpin structures of the partial novel miRNA precursors are shown in Additional file 2: Figure S1. MiRNAs are found on all 26 autosomes and chromosome X (Fig. 2), with marked differences in numbers among linkage groups. Although the distribution of miRNAs is highly similar in lambs and adults, miRNA expression differs between the two groups in chromosome 1, 2, 4, 5, 11, 15, 18 and 19.

### Differential expression of miRNAs between lambs and adults

**Fig. 3:**
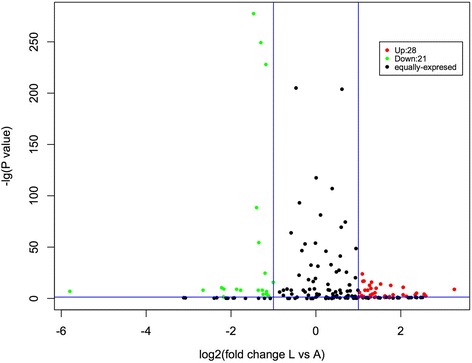
Analysis of differential miRNA expression between lambs and adults. Each point in the figure represents a microRNA. The X axis shows log_2_ normalized read counts and the Y-axis shows -log_10_(*p*-value). The vertical lines show thresholds for log_2_ ratio larger than 1 or lower than − 1. The horizontal line shows threshold for p-value< 0.05. The black points represent miRNAs exhibiting no significant differential expression between lambs and adults; the red points represent miRNAs that are more abundant in lambs than adults; the green points represent miRNAs that are less abundant in lambs than adults

In order to identify DE miRNAs, we compared the expression of miRNAs between the two lambs and two adults using the DESeq R package (1.8.3). The volcano plot in Fig. 3 summarizes the analysis result, and Additional file 3: Table S2 contains the list of DE miRNAs in detail. Forty-nine miRNAs exhibited differential expression between the L and A samples (|log2foldchange| > 1; qvalue< 0.01), of which 28 are more abundant, and 21 less abundant, in lambs than in adults (Table 2). Overall, the curly fleece trait in Tan lambs seems to be highly related to the expression of these miRNAs.

**Table 2:** Differentially expressed miRNAs between lamb (L) and adult (A) group

	miRNA	L_readcount	A_readcount	Log_2_ Fold Change	*P*-Value	P-Adj
Higher in lambs than in adults	***novel_138***	38.47793421	4.012949941	3.2613	1.57E-09	6.13E-09
	***oar-miR-1185-5p***	6.231432649	1.036267065	2.5882	0.0043264	0.0030268
	***novel_469***	12.13489516	2.072534129	2.5497	7.34E-05	5.96E-05
	***oar-miR-323c***	10.49504446	2.072534129	2.3402	0.0003234	0.00024917
	*oar-miR-544-5p*	17.36640855	3.835180153	2.1789	3.24E-05	1.71E-05
	*oar-miR-541-5p*	6.733913519	1.534072061	2.1341	0.010229	0.0040858
	*oar-miR-3959-3p*	47.84622764	11.50554046	2.0561	1.48E-11	1.01E-11
	*novel_468*	11.47895488	2.763378839	2.0545	0.00030638	0.00023908
	*oar-miR-376b-3p*	14.88549304	4.218698169	1.819	0.00033006	0.00016095
	*oar-miR-411b-5p*	63.79497018	18.79238275	1.7633	2.11E-13	1.49E-13
	*oar-miR-495-5p*	6.379497018	1.917590077	1.7341	0.021096	0.0080829
	*oar-miR-487a-3p*	8.527223625	2.763378839	1.6256	0.0040362	0.0028566
	*oar-miR-3955-5p*	64.61011747	22.45245307	1.5249	9.87E-15	1.25E-14
	*oar-miR-376c-3p*	58.47872267	21.86052687	1.4196	1.22E-10	7.90E-11
	*oar-miR-376e-5p*	7.08833002	2.684626107	1.4007	0.025836	0.009647
	*oar-miR-412-3p*	31.18865209	11.88905848	1.3914	3.13E-06	1.68E-06
	*oar-miR-758-3p*	24.10032207	9.587950383	1.3298	5.76E-05	2.96E-05
	*oar-miR-432*	102.7807853	41.41994566	1.3112	1.47E-16	1.17E-16
	*oar-miR-433-3p*	17.72082505	7.286842291	1.2821	0.00067931	0.00031883
	*oar-miR-487b-3p*	84.70554374	35.28365741	1.2635	1.53E-13	1.11E-13
	*oar-miR-410-3p*	53.16247515	22.24404489	1.257	5.30E-09	3.16E-09
	*oar-miR-379-5p*	4381.651202	1882.306419	1.219	0.00000001	0
	*oar-miR-127*	8086.012471	3631.532087	1.1549	0.00000001	0
	*oar-miR-380-3p*	124.4001919	56.76066627	1.132	1.18E-17	1.01E-17
	*oar-miR-409-5p*	124.7546084	57.91122032	1.1072	2.07E-17	1.69E-17
	*oar-miR-382-3p*	183.233331	85.90803543	1.0928	1.39E-24	1.37E-24
	*oar-miR-106a*	13.11880558	6.217602388	1.0772	0.0019904	0.0014596
	*oar-miR-539-3p*	42.17556362	20.70997283	1.0261	1.70E-06	9.38E-07
Lower in lambs than in adults	*oar-miR-30c*	2721.564311	5467.432827	−1.0064	1.96E-16	1.53E-16
	*oar-miR-191*	3111.422462	6305.803208	−1.0191	2.54E-20	2.27E-20
	*oar-miR-22-3p*	1979.061742	4076.796503	−1.0426	1.09E-15	8.36E-16
	*oar-let-7c*	5531.378331	11,531.23617	−1.0598	4.75E-46	6.14E-46
	*oar-miR-30a-3p*	158.0697595	342.8651057	−1.1171	0.0042634	0.0018614
	*oar-miR-133*	240.2943877	534.6241134	−1.1537	9.94E-05	5.04E-05
	*oar-miR-218a*	1060.327461	2385.141361	−1.1696	3.87E-05	3.23E-05
	*oar-let-7b*	14,204.65894	32,174.86038	−1.1796	1.20E-228	3.47E-228
	***novel_96***	31.10489768	73.83412387	−1.2471	0.00010598	0.00044248
	*oar-let-7a*	26,886.7446	64,467.46079	−1.2617	0.00000001	0
	*oar-miR-99a*	33,958.41705	81,484.92216	−1.2628	0.00000001	0
	*oar-miR-200c*	4132.423757	10,505.33008	−1.3461	4.02E-55	9.06E-55
	*oar-miR-26a*	56,450.87634	144,854.2462	−1.3595	0.00000001	0
	*oar-miR-26b*	5129.780951	13,523.97604	−1.3986	3.61E-89	9.15E-89
	*oar-miR-125b*	5760.685808	15,940.92631	−1.4684	3.36E-278	1.15E-277
	*oar-miR-29b*	101.0087028	342.8651057	−1.7632	3.74E-12	2.60E-12
	*oar-miR-148a*	67,574.96753	231,061.6487	−1.7737	0.00000001	0
	*oar-miR-136*	19.75893919	88.81995869	−2.1684	6.98E-10	2.81E-09
	***oar-miR-150***	50.50740147	235.578046	−2.2216	3.78E-11	4.18E-11
	***oar-miR-29a***	867.6115945	5480.855957	−2.6593	0.00000001	0
	***novel_459***	0	20.61202625	−5.3654	3.82E-06	1.82E-05

The DE miRNAs in boldface were validated with qRT-PCR

### Prediction of target genes and pathways analysis

**Fig. 4:**
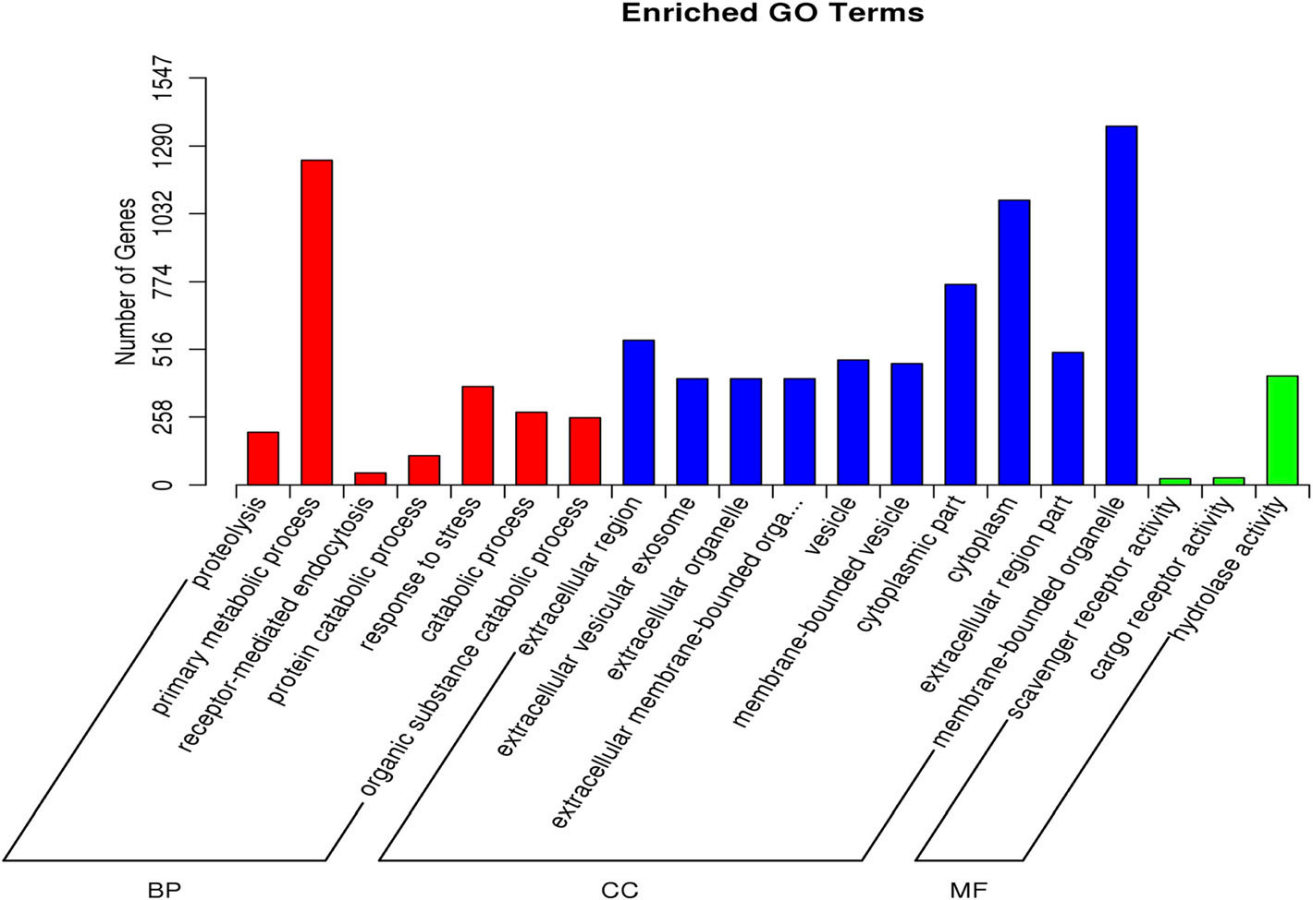
Gene Ontology (GO) analysis. 2363 genes, predicted to be targeted by the 28 miRNAs that were more abundant in lambs than in adults, were subjected to GO analysis. Each bar represents target gene number in each GO term in the category of biological process (BP), cellular component (CC) and molecular function (MF)

To better understand the biological function of the 49 DE miRNAs, their target genes were predicted using TargetScan (http://www.targetscan.org/vert_71/) and the NCBI Entrez database (https://www.ncbi.nlm.nih.gov/Class/MLACourse/Original8Hour/Entrez/). 2363 target genes were predicted for the 28 miRNAs that were more abundant in lambs (Additional file 4: Table S3A), and 2454 target genes were predicted for the 21 miRNAs that were less abundant in lambs (Additional file 4: Table S3B). In order to categorize the predicted target genes, a Gene Ontology (GO) enrichment analysis was performed. For those genes targeted by the miRNAs that were more abundant in lambs, 65 significant (*P* < 0.01) GO terms were identified, among which the most significant and highly enriched ones were cytoplasmic part, membrane-bounded vesicle, protein catabolic process, protein serine/threonine/tyrosine kinase activity, negative regulation of endothelial cell proliferation, extracellular membrane-bounded organelle, and primary metabolic process (Fig. 4, Additional file 5: Table S4A). These target genes were also classified using Kyoto Encyclopedia of Genes and Genomes (KEGG) functional annotations to identify the pathways in which they participate. The top 20 most commonly identified pathways (Table 3 and Additional file 6: Table S5A) include the metabolic MAPK and AMPK signaling pathways, which are known to be involved in hair growth and fleece morphology [23, 24]. In addition, we also analyzed the GO terms and KEGG pathways for target genes of miRNAs less abundant in lambs. The complete list of GO terms and pathways identified by the analysis is shown in Additional file 5: Table S4B and Additional file 6: Table S5B. The results showed that these genes are also enriched in the same pathways including MAPK and AMPK signaling pathways. These results proved that miRNAs either less or more abundant in lambs than in adults may contribute together to the formation of curly fleece in Chinese Tan lambs by regulating target genes involved in relative pathways.

**Table 3:** KEGG pathways enriched for target genes of the 28 miRNAs with higher expression in skin of lambs with curly fleece

KEGG pathway	Count	*P*-Value	Corrected *P*-Value
Metabolic pathways	180	0.894548594	0.984724863
Pathways in cancer	66	0.179111564	0.939563595
PI3K-Akt signaling pathway	64	0.39499336	0.939563595
HTLV-I infection	50	0.192543289	0.895292925
Phagosome	45	0.001758483	0.480065813
Protein processing in endoplasmic reticulum	43	0.007929522	0.545550536
Regulation of actin cytoskeleton	43	0.245615216	0.939563595
MAPK signaling pathway	42	0.630365388	0.939563595
Tuberculosis	41	0.051840788	0.750257819
Ras signaling pathway	41	0.474634575	0.939563595
Viral carcinogenesis	39	0.270545221	0.947474735
Transcriptional misregulation in cancer	37	0.104993737	0.779793658
Influenza A	37	0.185182388	0.939563595
Endocytosis	36	0.500604613	0.957112583
Epstein-Barr virus infection	35	0.270987612	0.947474735
Lysosome	33	0.01255486	0.638411076
MicroRNAs in cancer	33	0.443228539	0.939563595
Focal adhesion	33	0.531854202	0.957112583
cGMP-PKG signaling pathway	31	0.425371479	0.939563595
AMPK signaling pathway	30	0.071269601	0.779793658

### Integrated miRNA and mRNA analysis identifies target genes involved in curly fleece development

**Fig. 5:**
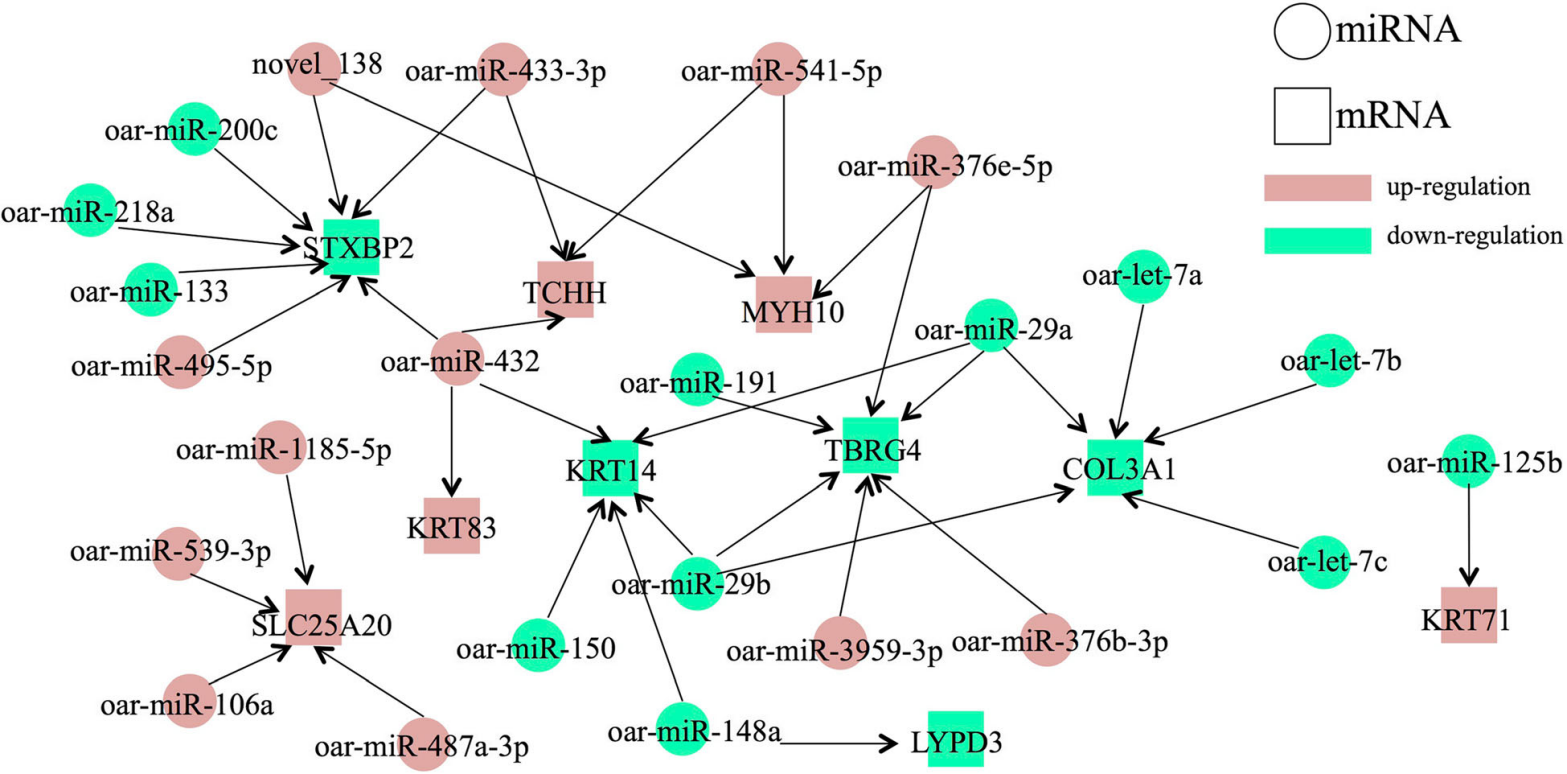
Integrated microRNA/mRNA network. The differentially expressed miRNAs and mRNA targets shown here were connected using TargetScan. Only miRNAs with the highest (red) or lowest fold changes (green) in lambs relative to adults are included

In order to generate a miRNA-mRNA interactome map, target genes of the DE miRNAs were searched in a list of differentially regulated mRNAs that had been generated in previous work [19]. The two data sets were analyzed using the miRanda algorithm [25], which identifies potential miRNA-mRNA functional interactions. All 49 differentially regulated miRNAs were used as input, but only target genes that were identified by both miRanda [25] and TargetScan (http://www.targetscan.org/vert_71/) (listed in Additional file 4: Table S3A and B, described above) were accepted for inclusion in the network. Thirty-six miRNA-mRNA pairs were identified, exhibiting both positive and negative correlation (Table 4). As shown in Fig. 5, 25 miRNAs (of which 12 were more abundant, and 13 were less abundant, in lambs vs. adults) were inversely correlated with 7 mRNA targets. In order to clarify the related functions of these miRNA regulated genes, a KEGG analysis was performed (Additional file 7: Table S6). The results showed that these targets are mainly associated with steroid biosynthesis and metabolic pathways, which have been known to regulate hormone production and skin and hair development [26]. Taken together, the results suggest that this miRNA/mRNA network may represent a key transcription network regulating curly fleece formation. Because only a few published studies have focused on curly fleece growth, and the current sheep expressed sequence tag (EST) data set is relatively sparse and incompletely annotated, additional members of the network may still be discovered in the future.

**Table 4:** List of miRNA-mRNA pairs and their expressions in lambs and adults

miRNAs			Target Genes	
Name	Sequence	Fold Change (Lamb vs. Adult)	Name	Expression in SSH
*novel_138*	UGGAUAACGCGUCUGACU	3.2613	*MYH10*	L > A
*novel_138*	UGGAUAACGCGUCUGACU	3.2613	*STXBP2*	L < A
*oar-miR-433-3p*	AUCAUGAUGGGCUCCUCGGUGU	1.2821	*STXBP2*	L < A
*oar-miR-433-3p*	AUCAUGAUGGGCUCCUCGGUGU	1.2821	*TCHH*	L > A
*oar-miR-541-5p*	AAAGGAUUCUGCUGUCGGUCCCACU	2.1341	*MYH10*	L > A
*oar-miR-541-5p*	AAAGGAUUCUGCUGUCGGUCCCACU	2.1341	*TCHH*	L > A
*oar-miR-1185-5p*	AGAGGAUACCCUUUGUAUGUUC	2.5882	*SLC25A20*	L > A
*oar-miR-150*	UCUCCCAACCCUUGUACCAGUG	−2.2216	*KRT14*	L < A
*oar-miR-3959-3p*	UGUAUGUCAACUGAUCCACAGU	2.0561	*TBRG4*	L < A
*oar-miR-376b-3p*	AUCAUAGAGGAAAAUCCAUGU	1.819	*TBRG4*	L < A
*oar-miR-495-5p*	AGAAGUCGCCCAUGUUCUUUUCG	1.7341	*STXBP2*	L < A
*oar-miR-487a-3p*	AUCAUACAGGGACAUCCAGUUU	1.6256	*SLC25A20*	L > A
*oar-miR-376e-5p*	GGUGGAUAUUCCUUCUAUGUUU	1.4007	*MYH10*	L > A
*oar-miR-376e-5p*	GGUGGAUAUUCCUUCUAUGUUU	1.4007	*TBRG4*	L < A
*oar-miR-432*	UCUUGGAGUAGGUCAUUGGGUGG	1.31	*KRT14*	L < A
*oar-miR-432*	UCUUGGAGUAGGUCAUUGGGUGG	1.3112	*TCHH*	L > A
*oar-miR-432*	UCUUGGAGUAGGUCAUUGGGUGG	1.3112	*KRT83*	L > A
*oar-miR-432*	UCUUGGAGUAGGUCAUUGGGUGG	1.3112	*STXBP2*	L < A
*oar-miR-106a*	AAAAGUGCUUACAGUGCAGGU	1.0772	*SLC25A20*	L > A
*oar-miR-539-3p*	AAUCAUACAAGGACAAUUUCUUU	1.0261	*SLC25A20*	L > A
*oar-miR-29a*	UAGCACCAUCUGAAAUCGGUU	−2.6593	*COL3A1*	L < A
*oar-miR-29a*	UAGCACCAUCUGAAAUCGGUU	−2.6593	*KRT14*	L < A
*oar-miR-29a*	UAGCACCAUCUGAAAUCGGUU	−2.6593	*TBRG4*	L < A
*oar-miR-148a*	UCAGUGCACUACAGAACUUUGU	−1.7737	*KRT14*	L < A
*oar-miR-148a*	UCAGUGCACUACAGAACUUUGU	−1.7737	*LYPD3*	L < A
*oar-miR-29b*	UAGCACCAUUUGAAAUCAGUGU	−1.7632	*COL3A1*	L < A
*oar-miR-29b*	UAGCACCAUUUGAAAUCAGUGU	−1.7632	*KRT14*	L < A
*oar-miR-29b*	UAGCACCAUUUGAAAUCAGUGU	−1.7632	*TBRG4*	L < A
*oar-miR-200c*	UAAUACUGCCGGGUAAUGAUGG	−1.3461	*STXBP2*	L < A
*oar-let-7a*	UGAGGUAGUAGGUUGUAUAGUU	−1.2617	*COL3A1*	L < A
*oar-let-7b*	UGAGGUAGUAGGUUGUGUGGU	−1.1796	*COL3A1*	L < A
*oar-let-7c*	UGAGGUAGUAGGUUGUAUGGUU	−1.0598	*COL3A1*	L < A
*oar-miR-218a*	UUGUGCUUGAUCUAACCAUGU	−1.1696	*STXBP2*	L < A
*oar-miR-133*	UUGGUCCCCUUCAACCAGCUGU	−1.1537	*STXBP2*	L < A
*oar-miR-125b*	UCCCUGAGACCCUAACUUGUG	−1.4684	*KRT71*	L > A
*oar-miR-191*	CAACGGAAUCCCAAAAGCAGCU	−1.0191	*TBRG4*	L < A

### qRT-PCR validation of selected miRNAs

**Fig. 6:**
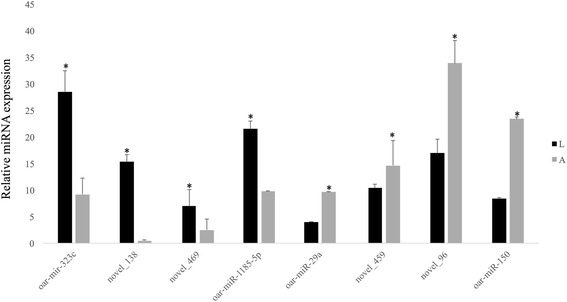
Validation of selected DE miRNAs through bulge-loop RT-qPCR technology. Each bar represents relative expression (mean ± SEM) of a miRNAs normalized by U6 small nuclear RNA in lambs (L) or adults (A). *indicates *p* < 0.05

To validate the results of miRNA-seq, expression levels of eight miRNAs were determined by qPR-PCR. Levels of *novel_138, oar-miR-1185-5p, novel_469* and *oar-miR-323c* were significantly higher in lambs than in adults, while levels of *novel_96, oar-miR-150, oar-miR-29a* and *novel_459* were lower in lambs (Fig. 6). These results are similar to those obtained using miRNA-Seq (Table 2). Although there were some quantitative differences between the two analytical platforms, the overall agreement suggests that the miRNA-Seq data are reproducible and reliable.

### *KRT83* was direct targets of *miR-432*

**Fig. 7:**
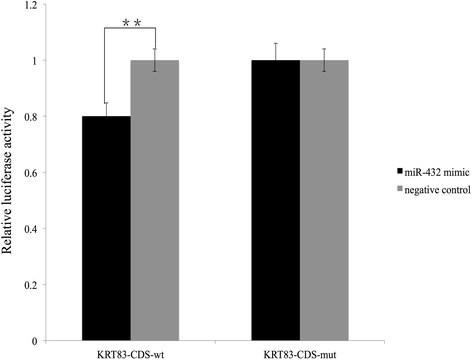
Hela cell culture transfections showing negative relationship between *oar-miR-432* and target *KRT83.* Cells were transfected with two plasmids containing wild type (wt) or mutant (mut) *KRT-83* CDS region, and then treated with *miR-432* mimic (black) or negative control (grey). Each bar represents relative luciferase activity (mean ± SEM) for triplicates of each transfection and treatment combination. ** indicates *p* < 0.01

Prediction online tools miRanda [25] and TargetScan (http://www.targetscan.org/vert_71/) detected a conserved *miR-432*-binding site in the CDS region of *KRT83* mRNA, we thus used dual luciferase reporter system to test whether *miR-432* could target *KRT83* expression (Fig. 7). As shown by dual-luciferase reporter assay in Hela cell line (Fig. 7), co-transfection of the Hela cell line with *miR-432* mimic or negative control did not induce any difference in luciferase activity of reporter in cells containing *KRT83*-CDS-mut plasmid, but induced quite different changes in luciferase activity in cells containing wild-type *KRT83*-CDS. When the cells with wild-type *KRT83*-CDS were treated with negative control or *miR-432*, *miR-432* mimic evidently decreased the luciferase activity compared to that in the cells treated by negative control. The result suggested that the previous prediction results were accurate.

**Fig. 8:**
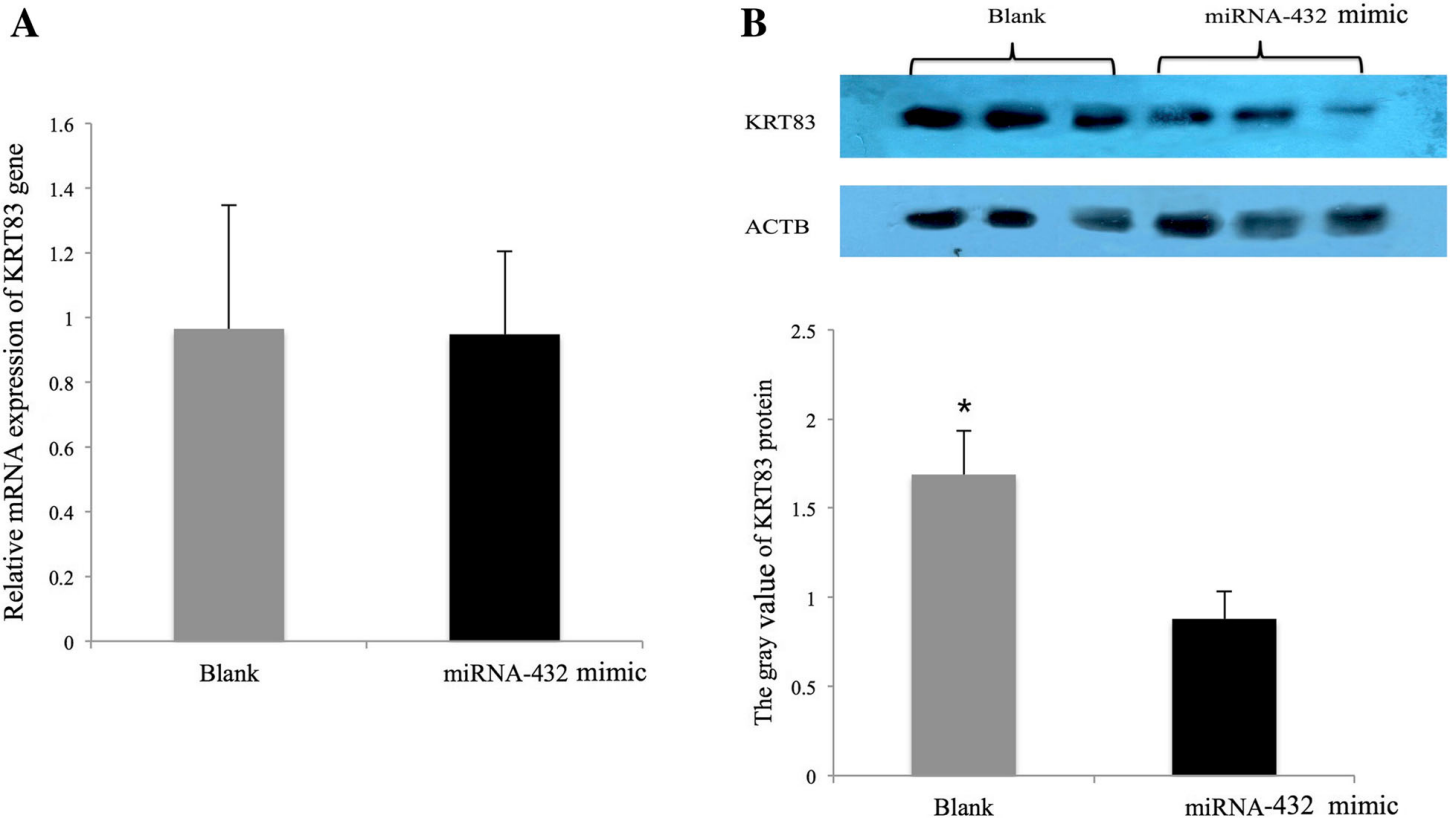
The overexpression of *miR-432* repressed the protein expression but not mRNA expression of KRT83 in primary sheep epidemial fiber cells. **a** mRNA expression of KRT83 show no significant different between cells treated by *miR-432* mimic and those treated by negative control miRNA. **b** Wester blotting showed decreased protein expression of KRT83 in cells treated by *miR-432* mimic compared to those treated by negative control miRNA. Each bar represents average gray value (mean ± SEM) of the bands in triplicates of each treatment

To further examine the effect of *miR-432* on expression of *KRT83* at both RNA and protein level, we transfected primary sheep epidermal fiber cells with *miR-432* mimic, and then compared the expression of *KRT83* in cells with that in cells treated by negative control miRNA. As shown in Fig. 8, after the same normalization with *ACTB* as housekeeping genes, qPCR showed no significant difference in *KRT83* mRNA expression between *miR-432* mimic and negative control treated cells, but western blotting showed significant decrease of KRT83 protein in cells treated by *miR-432* mimic compared to those treated by negative control.

## Discussion

miRNAs are important factors regulating skin and hair follicle development [27]. An investigation of miRNA expression in newborn mice showed that Dicer mRNA and multiple miRNAs are expressed in skin [28]. Through conditional knockout mutations in the essential microRNA biogenesis enzymes Drosha and Dicer, researchers showed that microRNAs are necessary to maintain the highly proliferative matrix cells in the hair follicle [29]. Mardaryev et al. were the first to demonstrate the significance of an individual microRNA as a regulator in hair follicle growth and function [30]. The relationship between miRNAs and target genes in the regulation of RNA transcripts is now established as a productive way for studying miRNA function [31].

In this study, we observed 49 DE miRNAs between lamb and adult Tan sheep, which may explain the fleece/hair growth and curly phenotypes. For example, members of the *let-7* family (*let-7a, let-7b* and *let-7c*) were detected to be less abundant in lambs with curly fleece than in adults with non-curling fleece, consistent with previous observation that *let-7* miRNAs showed decreased expression from small waves to medium waves and medium waves to large wave in Hu sheep [32]. Other studies also showed that *let-7* genes are related to the growth of hair follicles and hair quality in skin tissue [33, 34]. Therefore, we speculate that *let-7* miRNAs may function by altering the expression profiles in hair cells, thereby changing hair cell differentiation and function and facilitating the hair curvature. In addition, we also detected other miRNAs that are known to exhibit differential expression during the hair growth cycle, such as *oar-miR-125, oar-miR-127, oar-miR-200,* and *oar-miR-29* [35], and thus confirmed their involvement in hair development.

To understand the regulatory role of miRNAs in phenotypic variation of the curly fleece trait at two different growth stages, the targets of DE miRNAs were predicted to identify the key target genes regulated by key miRNAs. Functional analysis of the results with target genes showed significant enrichment for bio-functions related to curly fleece/hair growth. The top canonical pathway for target genes of miRNAs that were highly expressed in 1 month-old lambs sheep with curly fleece were enriched in the AMPK signaling pathway(*PFKFB1, FOXO3, GYS1, EEF2, RAB2A, SIRT1, HMGCR, PPP2CA, HNF4A, PPP2R5C, CPT1A, SCD, PIK3R1, PPP2R3C, RAB14, TSC2, PRKAG2, CPT1B, ADIPOQ, STRADA, ACACB, CAB39, ADRA1A, PFKL, CAB39L, PRKAA2*), Wnt signaling pathway(*PRKCG, DKK1, RUVBL1, DAAM1, CUL1, TBL1XR1, FZD3, CAMK2G, GSK3B, ROCK2, CSNK1A1, WNT5A, NFATC3, PPP3CA, APC, DVL2, FBXW11, FZD4, PLCB4, CAMK2A, protein Wnt-10b*) and MAPK signaling pathway (*PRKCG, FGF8, GADD45B, RPS6KA1, MAP2K2, TNF receptor-associated factor 2-like, MAP3K2, TRAF6, ELK4, ARRB1, MAP4K4, MAP3K5, NF1, FGF19, FAS, PLA2G4D, CASP3, PDGFRA, DUSP10, heat shock cognate 71 kDa protein-like, PAK2, MAPKAPK5, RASGRP2, PPP3CA, FGF7, RASGRF1, IL1B, MAPK3, ECSIT, CRK, HSPA8, NFATC3*). Among these target genes, *SIRT1* is implicated in the control of cell longevity, and it can prevent skin aging and hair loss and promote the cutaneous regeneration and hair growth. *SIRT1* was regulated by *oar-miR-29b*, *oar-miR-22-3p*, *oar-miR-23a* and *oar-miR-30a-5p*, which took part in the curly fleece growth [36]. *Wnt-10b* is another important gene for curly fleece growth. In C57BL/6 mice, *Wnt 10b* and *β-catenin* expression was through to be up-regulated along hair growth [37]. These two genes participate in not only regulation of hair follicle stem cell proliferation [38] but also the promotion of hair/fleece growth directly. Collectively, DE miRNAs and their target gene candidates assemble regulatory networks that may contribute to different phenotypes between different growth stages in Chinese Tan sheep.

To further study the regulation of miRNA-mRNA network in the process of curly fleece/hair formation, we integrated the data from miRNA-seq and SSH library. Then, 25 DE miRNAs and 10 DE target genes including *KRT83, KRT71, STXBP2, TCHH, MYH10, SLC25A20, KRT14, TBRG4, COL3A1* and *LYPD3* were found to form 36 miRNA-mRNA pairs (Fig. 5). Interestingly, the results from human study are consistent with our results, *KRT71* and *TCHH* have also been proposed as major genes whose variations affect curly hair growth in humans [4]. Especially, mutations of *KRT71* have been reported as the cause of wavy pelage in mice [39], wolly hair syndromes in humans [40], and curly hair in rats [41] and dogs [42]. Both *KRT71* and *TCHH* genes encode proteins in inner root sheath (IRS), which is a rigid structure in hair follicle strongly influencing hair shape by guiding shaft growth [43]. Therefore, the 25 DE miRNA may indirectly control IRS formation and hair growth by regulating expression of these major genes. These miRNA-mRNA pairs form a putative network where most of the miRNA expression patterns are negatively correlated with their target gene mRNA expression patterns and in agreement with previous reports [44]. For example, the *KRT71* gene is regulated by miRNAs *oar-miR-125b*. In this study, *KRT71* gene expression is 2.8 times lower [19] in q-PCR, but level of *oar-miR-125b* in miRNA-seq is more than 1.47 fold higher in adults relative to lambs (Table 4). However, there are also some miRNA-RNA pairs with positive correlation, such as *oar-miR-432* and *KRT83*. At the RNA level, expression of both *oar-miR-432* and *KRT83* was lower in adult than lamb. However, through cell culture experiments, we found that protein expression of *KRT83* gene was still negatively by *oar-miR-43,* indicating variable impact of miRNA on post-transcription regulation as well as possible positive feedback loop of *KRT83* RNA expression and degradation. On the other hand, due to the limited sample size in this miRNA-seq study and possible difference between in vivo and in vitro gene expression regulation, we also could not exclude the possibility of bias that may affect our observation and conclusion.

As our survey of putative sheep miRNAs was performed using samples derived from skin tissues containing a wide variety of cell types, our analyses may have failed to detect some miRNAs and mRNAs with low copy number but specific to dermal papilla cells and the induction of curly fleece. In spite of this limitation, skin tissue-derived mRNA and miRNAs were detected with high sensitivity, and specific markers for curly fleece growth were successfully characterized. Because skin samples are readily available and requiring minimal handling during sample collection, they are ideal for monitoring miRNA expression in hair and wool development. Overall, the regulatory network and miRNA/mRNA components described here are a starting point for understanding of the mechanisms underlying curly fleece development in Tan lambs. Moreover, the high overall degree of similarity between mammals skin tissues [19] make the sheep an attractive model for basic research of hair and wool growth in humans.

## Conclusions

This study explores the role of miRNAs in the curly fleece trait of Chinese Tan sheep. Several DE miRNAs and potential mRNA targets were identified by comparing high-throughput miRNA and mRNA sequence data from lambs and adults. Two hundred and thirty two skin-specific miRNAs were found, of which 49 are differentially expressed. Target analysis implicated several key signaling pathways (e.g., MAPK, WNT and AMPK) in curly fleece formation. After integrating miRNA-seq and mRNA-seq data, 36 putative miRNA-mRNA pairs were identified (Table 4). Most miRNA-mRNA pairs exhibit negatively correlated expression patterns, but about 25% of the pairs are positively correlated. The 36 pairs appear to constitute a complex regulatory network which functions in steroid biosynthesis, metabolic pathways, and the Wnt, AMPK, and MAPK signaling pathways. This network is highly likely to be involved in the development of curly fleece in Tan lambs and its disappearance in adult sheep. Our results provide important clues for elucidating the molecular mechanism underlying curly fleece and curly hair development.

## Methods

### Sample preparation and total RNA extraction

Animal care and the experiments were conducted according to the Regulations for the Administration of Affairs Concerning Experimental Animals (Ministry of Science and Technology, China, revised in June 2004) and approved by the animal welfare committee of the State Key Laboratory for Agro-biotechnology of China Agricultural University (approval number XK257). Four female Chinese Tan sheep (two 1-month-old lambs and two 48-month-old adults) at Tan sheep farm in Yinchuan (located at Ningxia province, China) were randomly selected but have no relationship between each other and were raised under the same conditions in this study, which has been permitted by the owner. The hair and growth phenotypes are similar between two individuals in each group, when they were humanely slaughtered by the farm owner. Then, skin tissue was collected from the shoulder of each carcass and immediately frozen in liquid nitrogen or at − 80 until use, and total RNA was extracted using TRIzol reagent (Invitrogen, CA, USA) following the manufacturer’s instructions. RNA quality and quantity were assessed using a Nanodrop 2000 spectrophotometer (Thermo Fisher Scientific, MA, USA). RNA samples were stored at − 80 °C.

### Library preparation for small RNA sequencing

Four RNA samples were used to construct two small RNA libraries, one containing two lamb samples, and one containing two adult samples. Each sample contributed 3 μg RNA to the corresponding library. Sequencing libraries were generated using the NEBNext® Multiplex Small RNA Library Prep Set for Illumina® (NEB, USA), following the manufacturer’s recommendations. Indexing (barcoding) was used to distinguish each sample. Briefly, NEB 3’ SR adaptor was first ligated to the RNA 3′ ends. After the ligation reaction, SR RT primer was annealed to the 3’ SR adaptor (including any free adaptor remaining after the 3′ ligation reaction) to generate double-stranded molecules consisting of SR RT primer - 3’ SR adaptor hybrids. The 5′ end adaptor was then ligated to 5′ end of the preparation using T4 RNA Ligase 1. Importantly, this enzyme does not accept dsDNA as a substrate, and therefore the primer-adapter hybrids formed in the previous step cannot ligate to generate adapter dimers. First strand cDNA synthesis was accomplished using M-MuLV Reverse Transcriptase (RNase H–). PCR amplification was then performed using LongAmp Taq 2X Master Mix, SR primer for Illumina, and the appropriate index (X) primer. PCR products were separated on an 8% polyacrylamide gel (100 V, 80 min). DNA fragments with length of 140~ 160 bp (the length of a typical small noncoding RNA plus the 3′ and 5′ adaptors) were recovered with QIAquick Gel Extraction Kit (Qiagen, Beijing, China) following the manufacturer’s instruction and dissolved in 8 μL elution buffer. Library quality was assessed on the Agilent Bioanalyzer 2100 system using DNA High Sensitivity Chips (Novogene).

### Data analysis and miRNA annotation

Sequence data were generated using the Illumina HiSeq 2500/2000 platform. Reads were filtered out with NGQC software (Novogene) if they met any of the exclusion criteria (N% > 10, quality score < 50%, 5′ adaptor contamination, 3′ adaptor absence, missing insert, contained poly-A, G, C, or T.) Clean reads of 15~ 35 bp in length were used for further analysis. Sequences matching the sheep reference genome (oar_v3.1; http://www.livestockgenomics.csiro.au/sheep/oar3.1.php) were identified, and tags matching protein-coding sequences were eliminated from the data set. To remove tags corresponding to low-complexity sequences including rRNA, tRNA, snRNA, and snoRNA, reads were screened using RepeatMasker (http://www.repeatmasker.org) and compared with entries in the Rfam database (http://rfam.xfam.org). The remaining sequences were considered as candidate miRNAs. With miRBase as a reference (http://www.mirbase.org), we used a modified version of mirdeep2 [22] (https://www.mdc-berlin.de/8551903/en/) and srna-tools-cli (http://srna-tools.cmp.uea.ac.uk/) to predict secondary structures. miREvo [21] and mirdeep2 [22] were then used to predict novel miRNAs by examining characteristics of the secondary structures, the Dicer cleavage sites, and the minimum free energy of the folded models.

Differential expression analysis of the lamb and adult data was performed using the DESeq R package (1.8.3). MiRNA counts were determined using custom scripts. *P*-values were adjusted using the Benjamini & Hochberg [45] method. A corrected P-value of 0.05 (default value) was used as the significance threshold for differential expression. MiRNA expression levels were estimated by TPM using the normalization formula [46]: Normalized expression = mapped readcount/Total reads*1000000.

### Target genes prediction and pathway and gene ontology analyses

To predict the genes targeted by DE miRNAs, online tools miRanda [25] and TargetScan (http://www.targetscan.org/vert_71/) was used to identify potential miRNA binding sites.

With the predicted target genes, we performed KEGG pathways analysis using KOBAS [47] application to determine enrichment of these genes in certain pathway. Target gene candidates were also subjected to GO enrichment analysis. The GOseq-based Wallenius non-central hyper-geometric distribution [48], which adjusts for gene length bias, was used to determine GO enrichment significance.

### miRNA identification by bulge-loop RT-qPCR

To validate the expression of mature miRNAs, miRNA expression was quantified in individual samples using a Bulge-Loop reverse-transcription quantitative polymerase chain reaction (RT-qPCR) assay (RiboBio, Guangzhou, China). One reverse transcription primer and a pair of quantitative PCR primers were designed for each of miRNAs including *novel_138, oar-miR-1185-5p, novel_469, oar-miR-323c, novel_96, oar-miR-150, oar-miR-29a,* and *novel_459*. Reverse transcription of total miRNA was conducted using 1 μg total RNA per sample and the QuantiTect Reverse Transcription Kit (Qiagen). Real-time quantitative-PCR was carried out using SYBR Green qPCR mix (Tiangen, Beijing, China), with U6 small nuclear RNA as an internal reference for normalization. Quantitative-PCR reactions were performed using a BioRad CFX96 (BioRad, CA, USA). Relative miRNA expression was calculated using the standard curve-based method for relative real-time PCR, which has been previously described [25].

### Integrated miRNA-mRNA-analysis

In order to identify positive and negative relationships between miRNA and mRNA expression, we used our previous SSH data to integrate with the DE miRNAs and scanned for potential target genes. Only miRNA genes exhibiting correlated expression with their targets were included in the network visualization analysis. Pearson correlation of miRNA and mRNA expression levels was calculated.

### Luciferase reporter assays

The targets sequence of the predicted *miR-432* binding site that includes CDS region of *KRT83* gene were cloned from sheep genomic DNA and inserted into the psi-CHECK2 plasmid (Promega) using AsiI and pmeI restriction sites downstream from the Renilla luciferase gene. To validate binding of *miR-432* with *KRT83,* another primer was subsequently synthesized by Sangon (Shanghai, China) to introduce mutation of some bases at the 22 bp putative binding site of *miR-432* in *KRT83*. All primers used are listed in Additional file 8: Table S7.

### Cell culture and transfection

Primary sheep epidermal fiber cells and HeLa cell lines were purchased from Xiehe Medical University (Beijing, China). The cells were grown overnight in 24-well plates at concentration of 10 [5] cells/well in DMEM supplemented with 10% FBS (Hyclone, Gibco). Plasmids containing *KRT83* CDS region were co-transfected with either *miR-432* or negative control miRNA to each well at a concentration of 100 nM in triplicates (Lipofectamine, Invitrogen). Cells were harvested 48 h post-transfection and luciferase activity was measured using the Dual-Glo™ Luciferase Assay System (Promega, Beijing, China) according to the manufacturer’s instructions. Data was first normalized per-well by taking the ratio of Renilla luminescence (CDS region) to Firefly luminescence (transfection control). The ratio of the mean of the three biological replicates for each *miR-432* transfected group to the mean of the corresponding control siRNA transfected group was calculated as the relative luciferase activity.

For primary sheep epidermal fiber cells, *miR-432* mimic (100 nM) was transfected into cells using Lipofectamine™ 2000 (Invitrogen), according to the manufacturer’s protocol, and cells were harvested 48 h post-transfection using for RNA and protein extraction.

### Western blotting

Cells were lysed in RIPA lysis buffer on ice for 30 min with a protease inhibitor cocktail (Huitiandongfang, China), and centrifuged at 12000*g for 1 h. Then cell lysates were collected and the protein concentration was measured. Proteins (30μg) were separated by 10% polyacrylamide gel electrophoresis and then transferred onto PVDF membranes (Bio-Rad, Hercules, CA). The membranes was incubated with blocking liquid at 4 °C overnight, and then incubated with the primary antibodies against KRT83 (1/3000, Abcam) and beta-tublin (1/5000, Santa Cruz) for 2 h at room temperature. Membranes were washed, incubated with HRP-conjugated secondary antibody for 1 h and in blocking solution (1/5000, Santa) at room temperature, and wash again. The proteins signals were detected using Pierce ECL Western Blotting Substrate kit (Thermo Scientific™) according to the manufacturer’s instructions in dark room. Gray value of each target band in the western blotting was measure using Image J software.

### Statistics

Data are expressed as means ± standard deviation. Experiments were repeated at least twice, and each experiment included at least three replicates. Data from different treatments were subjected to an analysis of variance (ANOVA), and means were compared using Duncan’s multiple range test. All statistical analyses were performed using SPSS 16.0 (IBM Corporation). Differences were deemed statistically significant if *p*-values are smaller than 0.05.

## Additional files

Additional file 1: **Table S1.** Conserved and novel miRNAs detected in lambs and adults. (XLS 82 kb)

Additional file 2: **Figure S1.** Secondary structure predictions for novel miRNAs. The red color shows the mature miRNA sequences. (TIFF 19777 kb)

Additional file 3: **Table S2.** miRNAs exhibiting differential expression between the skin samples of lambs and adults. (XLS 39 kb)

Additional file 4: **Table S3.** Predicted target genes of differentially regulated microRNAs in lambs and adults (Table S3A: Target genes for the 28 miRNAs more abundant in lambs than in adults; Table S3B: Target genes for the 21 miRNAs less abundant in lambs than in adults). (ZIP 383 kb)

Additional file 5: **Table S4.** Detailed results of GO terms analysis for gene transcripts targeted by miRNAs (Table S4A showed the GO terms of target genes of the miRNAs that are more abundant in lambs than in adults; Table S4B showed the GO terms of target genes of the miRNAs that are less abundant in lambs than in adults). (XLS 922 kb)

Additional file 6: **Table S5.** Signaling pathways revealed by KEGG analysis that involve miRNAs highly expressed in lambs and their target genes (Table S4A showed pathways involving target genes of the miRNAs that are more abundant in lambs than in adults; Table S4B showed pathways involving target genes of the miRNAs that are less abundant in lambs than in adults). (XLS 254 kb)

Additional file 7: **Table S6.** The signaling pathways analysis with target genes that form miRNA-mRNA pairs with differentially expressed miRNAs between the two stages of Tan sheep. (XLS 30 kb)

Additional file 8: **Table S7.** Sequences of primer used for amplification of wild type or mutant KRT83 CDS region. (DOCX 48 kb)

## Abbreviations

DE: Differentially expressed
GO: Gene Ontology
KEGG: Kyoto Encyclopedia of Genes and Genomes |EST Expressed sequence tag; IRS: inner root sheath
miRNA: microRNA;
SSH: Suppression Subtractive Hybridization
