# Supplementary figures and images for: Integrated miRNA-mRNA analysis reveals regulatory pathways underlying the curly fleece trait in Chinese tan sheep
Source: BMC Genomics. 2018 May 11;19:360. doi: 10.1186/s12864-018-4736-4 (PMC5948824; doi:10.1186/s12864-018-4736-4)

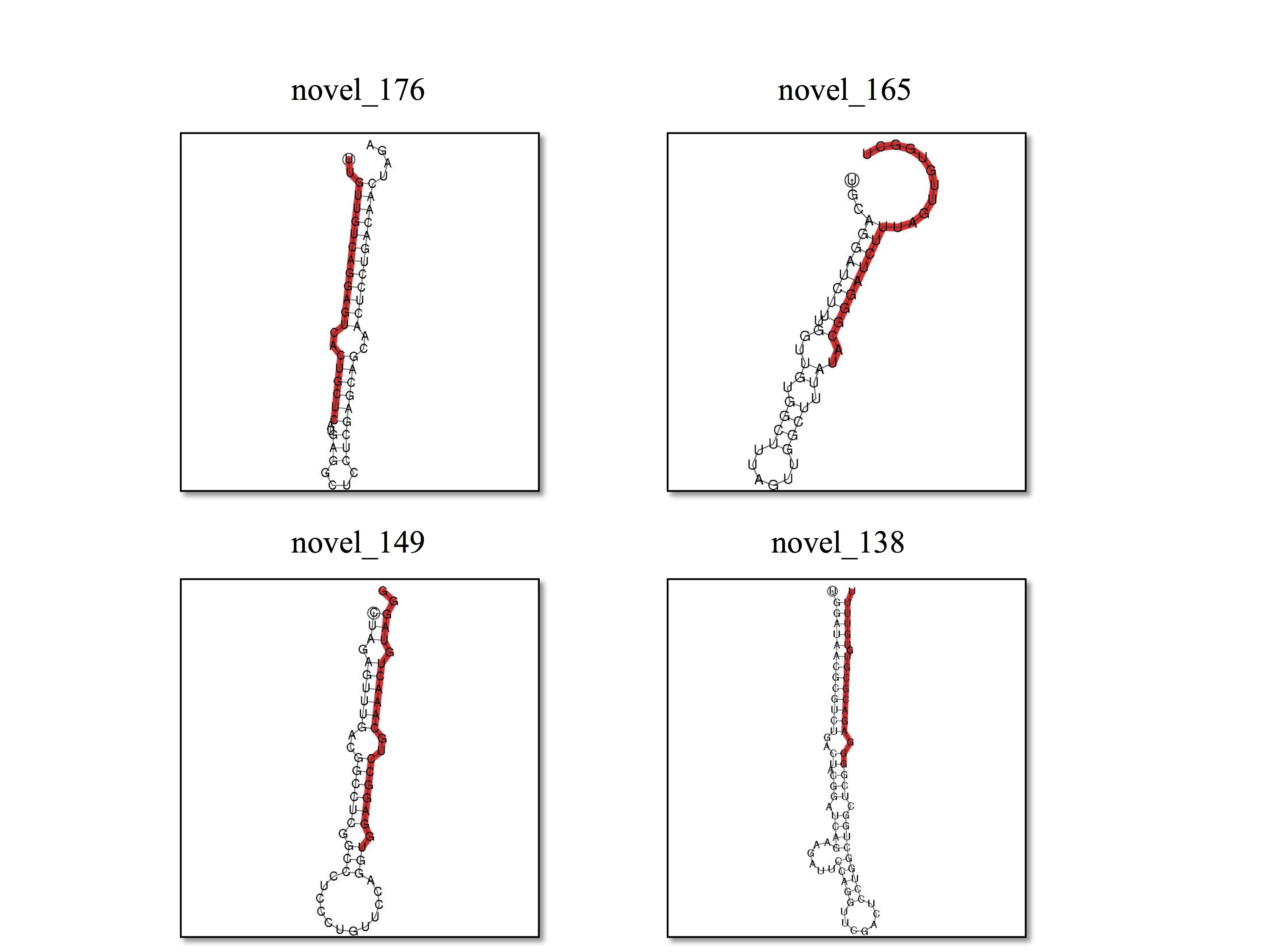

Supplement: Supplementary file 2 — Figure S1. Secondary structure predictions for novel miRNAs. The red color shows the mature miRNA sequences. (TIFF 19777 kb) [file 12864_2018_4736_MOESM2_ESM.tiff]
