# Supplementary material for: Integrated miRNA-mRNA analysis reveals regulatory pathways underlying the curly fleece trait in Chinese tan sheep
Source: BMC Genomics. 2018 May 11;19:360. doi: 10.1186/s12864-018-4736-4 (PMC5948824; doi:10.1186/s12864-018-4736-4)
Supplement: Supplementary file 8 — Table S7. Sequences of primer used for amplification of wild type or mutant KRT83 CDS region. (DOCX 48 kb) [file 12864_2018_4736_MOESM8_ESM.docx]

| **Name** | **Primer sequence (5’ to 3’)** |
| --- | --- |
| F | ATTGCGATCGCCAATGAGCTGAACCGCGTGATCCAGAGGCTGACAGCTGAGGTTGAGAATGCCAAGTGCC |
| R | TAAGTTTAAACCAGCCTGGGTGACTGCAGCCTCCAGCTTGGAGTTCTGGCACTTGGCATTCTCAACCTC |
| MUT | TAAGTTTAAACCAGCCTGGGTGACTGCAGCCTCCAGGAACCTCATCTGGCACTTGGCATTCTCAACCTC |

**Table S7: Sequences of primer used for amplification of wild type or mutant KRT83 CDS region.**

Note: The red letters indicate the miRNA binding sites and introduced mutation sites.
